# Clinical impact of removing respiratory motion during liver SABR

**DOI:** 10.1186/s13014-019-1300-6

**Published:** 2019-06-03

**Authors:** M. Gargett, C. Haddad, A. Kneebone, J. T. Booth, N. Hardcastle

**Affiliations:** 1 Northern Sydney Cancer Centre, Royal North Shore Hospital, Sydney, NSW Australia; 2 Institute of Medical Physics, University of Sydney, Sydney, NSW Australia; 3 Department of Physical Sciences, Peter MacCallum Cancer Centre, East Melbourne, VIC Australia

**Keywords:** Liver SABR, Liver SBRT, Motion management, Dose escalation

## Abstract

**Background:**

Liver tumors are subject to motion with respiration, which is typically accounted for by increasing the target volume. The prescription dose is often reduced to keep the mean liver dose under a threshold level to limit the probability of radiation induced liver toxicity. A retrospective planning study was performed to determine the potential clinical gains of removal of respiratory motion from liver SABR treatment volumes, which may be achieved with gating or tumor tracking.

**Methods:**

Twenty consecutive liver SABR patients were analysed. The treated PTV included the GTV in all phases of respiration (ITV) with a 5 mm margin. The goal prescription was 50Gy/5# (BED 100 Gy_10_) but was reduced by 2.5 Gy increments to meet liver dose constraints. Elimination of motion was modelled by contouring the GTV in the expiration phase only, with a 5 mm PTV margin. All patients were replanned using the no-motion PTV and tumor dose was escalated to higher prescription levels where feasible given organ-at-risk constraints. For the cohort of patients with metastatic disease, BED gains were correlated to increases in tumour control probability (TCP). The effect of the gradient of the TCP curve on the magnitude of TCP increase was evaluated by repeating the study for an additional prescription structure, 54Gy/3# (BED 151 Gy_10_).

**Results:**

Correlation between PTV size and prescribed dose exists; PTVs encompassing < 10% of the liver could receive the highest prescription level. A monotonically increasing correlation (Spearman’s rho 0.771, *p* = 0.002) between the degree of PTV size reduction and motion vector magnitude was observed for GTV sizes <100cm^3^. For 11/13 patients initially planned to a decreased prescription, tumor dose escalation was possible (5.4Gy_10_–21.4Gy_10_ BED) using the no-motion PTV. Dose escalation in excess of 20 Gy_10_ increased the associated TCP by 5% or more. A comparison of TCP gains between the two fractionation schedules showed that, for the same patient geometry, the absolute increase in BED was the overarching factor rather than the gradient of the TCP curve.

**Conclusions:**

In liver SABR treatments unable to be prescribed optimal dose due to exceeding mean liver thresholds, eliminating respiratory motion allowed dose escalation in the majority of patients studied and substantially increased TCP.

## Background

Primary liver cancer presents a large cancer burden, with an estimated 745,000 deaths globally per year [1]. Moreover many cancers metastasise to the liver; it is estimated that up to 50% of colorectal cancers and 25% of breast cancers will develop liver metastases [2, 3]. Surgical resection is currently the gold standard treatment for cancers in the liver, however it is often only appropriate in the minority of cases [4, 5]. Where comorbidities exclude resection as an option, less invasive approaches are sought, such as Stereotactic Ablative Body Radiotherapy (SABR). SABR has been shown to have equivalent rates of local control and quality of life indicators to other minimally-invasive treatment alternatives such as radioembolisation with selective internal radiotherapy, transarterial chemoembolization and radiofrequency ablation [6].

SABR delivers highly conformal distributions of ablative dose to the target volume; some surrounding healthy liver however is almost always included in the radiation field. As the liver undergoes motion with respiration, radiation therapy treatment requires the inclusion of this motion in planning target volume (PTV) margins through the use of an internal target volume (ITV) or through mid-ventilation margin generation. This results in exposing more of the liver volume, or gastrointestinal (GI) structures, to ablative doses of radiation to avoid a geographic miss. Radiation induced liver disease (RILD), and GI toxicities, are known complications of SABR treatments [7–11]. Consequently, isotoxic prescription regimes are used to limit dose to the liver and GI structures. Employing advanced motion management techniques such as respiratory gating in breath-hold or free-breathing, as well as real time tracking can potentially reduce the treated volume of liver and GI structures.

Limiting the decrease in dose from isotoxic prescription can directly impact the success of liver SABR treatments, in particular for cases of metastatic disease [12–14]. In the liver-organ-specific Hypofractionatioed Treatment Effects in the Clinic (HyTEC) report from the American Association of Physicists in Medicine Working Group on Biological Effects of Hypofractionation, an analysis of local control rates following liver SABR was compiled based on a collection of publications forming a sample size of 721 tumours of both primary (431) and metastatic (290) origin [14]. For metastatic disease there was found to be a relationship between local control and the delivered biologically effective dose (BED), with one-, two- and three- year local control rates being significantly higher for BEDs > 100 Gy_10_ compared to treatments of lower doses. The 2 year local control rate increased from 70 to 93% for BEDs > 100 Gy_10_. For primary tumours there were high local control rates at relatively low SABR doses, with no indicated relationship between local control and dose for BEDs 60–180 Gy_10_. As such, aggressive treatment regimens are recommended for metastatic disease, while more standard 40–50 Gy in 5 fraction prescriptions are sufficient for primary disease.

The above-mentioned technological advancements in radiation therapy delivery, such as respiratory-gated deliveries and tumor tracking [15–20], have the potential to further improve clinical outcomes by reducing the treated volume. This work quantifies the reductions in the amount of healthy liver in the treatment volume, and the increase in tumor BED facilitated by subsequent mean liver dose reductions. We retrospectively replanned 20 liver SABR patients with zero motion contribution to the PTV margin to simulate respiratory gating or tumor tracking techniques. Comparison was drawn to the original ITV-based treatment plans to assess the potential for target dose escalation as a direct result of dose sparing to prescription-limiting normal tissue structures. As indicated by the literature, BED gains for oligometastatic disease have a strong correlation with local control rates [12, 14]. In consideration of this, TCP gains resultant from target dose escalation are presented for the metastatic cohort.

## Methods

All patients receiving SABR to primary liver cancer or liver metastases in our institution over a 3 year period were reviewed for inclusion. The achievable prescription doses in an isotoxic prescription regime were analysed for all patients in this period. A subset of patients were selected for a retrospective treatment planning study to evaluate the impact of tumor motion on achievable prescription dose and organ at risk doses. Inclusion criteria for the planning study included visible tumors that were imaged using a free-breathing 4D-CT.

Volumetric modulated arc therapy (VMAT) was used, planned with the Eclipse (Varian Medical Systems, Palo Alto) treatment planning system with the Anisotropic Analytical Algorithm (v11.0.31) for dose calculation and Photon Optimization algorithm (v13.5.35) for optimization. Plans were optimised to adhere to the target coverage and organ at risk (OAR) dose constraints recommended by the RTOG1112 liver SABR trial protocol [21] for a 5 fraction treatment schedule; 100% of the prescription dose was to cover at least 95% of the target volume and the allowed mean liver dose (excluding the GTV) should not exceed 13–16 Gy, depending on the effective liver volume irradiated. The starting prescription level was 50 Gy.

Patients included in the study were simulated with a free-breathing 4D-CT scan. The 4D-CT was acquired using the Bellows system (Phillips Medical Systems, Cleveland, USA), a pneumatic belt, to sort the scan into 10 phase-based bins. Venous contrast was used during the simulation 4D-CT to aid contouring for metastatic lesions. For each patient, the motion of each lesion during the respiratory cycle was quantified by calculating the magnitude of the 3D motion vector of the tumor centre of mass between end-inspiration and end-expiration.

An ITV method was used to create a PTV margin that included motion of the tumor through the respiratory cycle. The extent of anterio-inferio-lateral movement of liver tumors was accounted for by contouring the gross tumor volume (GTV) on the end-inspiration and end-expiration phase images respectively and summing them on the average image set reconstructed from the 4D-CT to create the ITV. The ITV contour was reviewed on all intermediate phases and edited where necessary to ensure tumor coverage during the entire respiratory cycle. The PTV was a uniform 5 mm expansion of the ITV. This PTV will be referred to as the ‘ITV-based’ PTV for the remainder of this manuscript.

A ‘motion managed’ PTV was created for each plan, that does not include tumor motion with respiration. It is a 5 mm expansion on the GTV contoured on the exhale phase of the 4D-CT; that is, the same magnitude PTV expansion that was applied to the ITV-based plan to account for random and systematic uncertainty. The exhale phase was chosen because it is considered the most anatomically stable, and often has the longest duty cycle of all the phases. The motion managed PTV represents the theoretically smallest achievable PTV if one were able to remove all respiratory motion.

A motion managed plan was created for all patients by re-optimising the ITV-based plan (on the exhale phase image set) using the motion managed PTV as the target structure. The beam arrangement (number of arcs, start-stop angles and collimator angle) was not modified in the process. The motion managed plan was accepted when it met the PTV coverage and OAR constraints prescribed to the ITV-based plan.

For patients treated to a decreased dose prescription with the ITV-based plan, possible dose escalation to the target was evaluated in the context of organ at risk doses. An additional motion managed plan was created that was iteratively optimised to higher prescription levels until the maximum prescription dose was reached, or was limited by OAR constraints.

The cohort of patients with metastatic disease were planned to an additional commonly used SABR prescription; a 3 fraction schedule with a starting prescription dose level of 54 Gy. The procedure for dose escalation described earlier was replicated. The Institute of Cancer Research’s COnventional care versus Radioablation for Extracranial oligometastases (CORE) [22] trial protocol OAR constraints were applied. The dose constraints to the liver differ to the RTOG1112 liver SABR trial protocol [21]; in place of a mean dose constraint of 13 Gy to the liver were D_50%_ < 15 Gy and D_700cm_^3^ < 15 Gy constraints.

The purpose of including the additional prescription schedule was twofold; firstly, dose escalation for this particular prescription structure is of interest to the metastatic cohort due to evidence of improved local control rates for BED > 100 Gy_10_ [12–14] (a 54Gy/3# regimen equates to a BED of 151.2 Gy_10_). Secondly, BED escalation for the additional prescription schedule effectively extended the range of analysis within the TCP curve, which enabled evaluation of the magnitude of TCP gains in relation to the slope of the TCP curve. Increases in TCP due to escalation of BED in this 3 fraction schedule are presumed to be less than for the 5 fraction schedule (BED 100 Gy_10_), because this BED range sits closer to the shoulder of the curve. The respective slopes of the tangents to the TCP curve at BED 100 Gy_10_ (5 fraction) and BED 151.2 Gy_10_ (3 fraction) are 0.26%/Gy_10_ and 0.17%/Gy_10_.

BED was calculated for each patient case using the Linear Quadratic Equation, based on the prescribed dose regimen and α/β = 10 Gy. The corresponding TCP was calculated using the model parameters published in the HyTEC Organ-Specific Paper regarding liver TCP [14]. Here TCP refers to the rate of local control at 24 months. The model parameters were determined through analysis of published local control rates and are directly applicable to liver SABR.

Where statistical correlations of datasets are presented a Spearman’s rho was calculated, with an associated *p*-value to indicate the significance of the correlation. A Spearman’s rho close to + 1.0 indicates a strong monotonically increasing relationship. The calculation of a Spearman’s correlation was chosen over a Pearson’s due to the preference towards identifying monotonically increasing relationships between variables, rather than strictly linear relationships. The *p*-value specifies whether the Spearman’s rho value is significantly different from 0. Spearman’s rho and *p*-values were calculated using the Statistics and Machine Learning Toolbox in MATLAB v.2017a.

## Results

### Cohort data

**Fig. 1:**
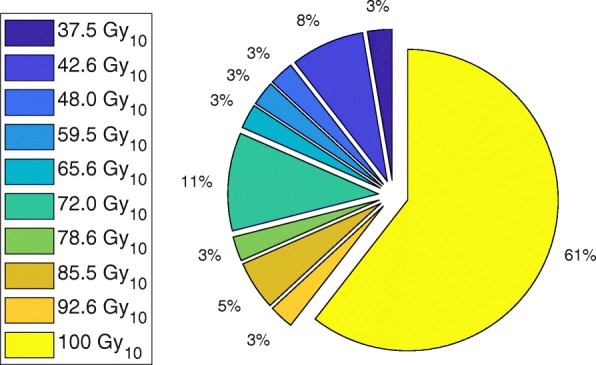
The percentage of patients treated at each prescription level at our institution (38 cases in total), without motion management

At our institution, 38 patients were treated with SABR for primary (10/38) and metastatic (28/38) liver disease over the 3 year period considered in this study. Figure 1 shows the percentage of patients treated at each prescription level, presented in terms of BED covering 95% of the PTV. The maximum total dose level used in our institution is 50 Gy, delivered over 5 fractions, corresponding to a BED of 100 Gy_10_. In 39% of the 38 cases, it was necessary to decrease the prescription level due to unacceptably high dose in the surrounding liver or nearby gastrointestinal structures such as the duodenum and oesophagus. The minimum total dose prescribed was 25 Gy in 5 fractions (37.5 Gy_10_). Of the 38 cases, 23 tumors in 20 patients could be visualised on the treatment planning 4D-CT and were selected for the planning study.

**Fig. 2:**
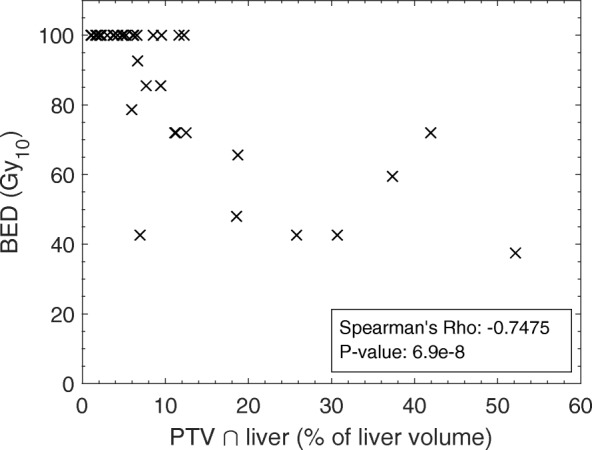
Distribution of PTV size with prescribed BED for 38 patients. A monotonically decreasing relationship is observed, with Spearman’s rho 0.7475 (*p* = 6.9 × 10^− 8^)

Figure 2 shows prescribed BED as a function of the percentage of the liver volume encompassed by the PTV for the 38 patients included in this study. Typically only PTVs encompassing less than approximately 10% of the liver could receive the highest prescription level of 100 Gy_10_ BED. A decrease in BED was observed as PTV size increased, indicating a reduction in PTV–liver overlap may facilitate escalation of dose to the target.

### PTV reduction with motion management

**Fig. 3:**
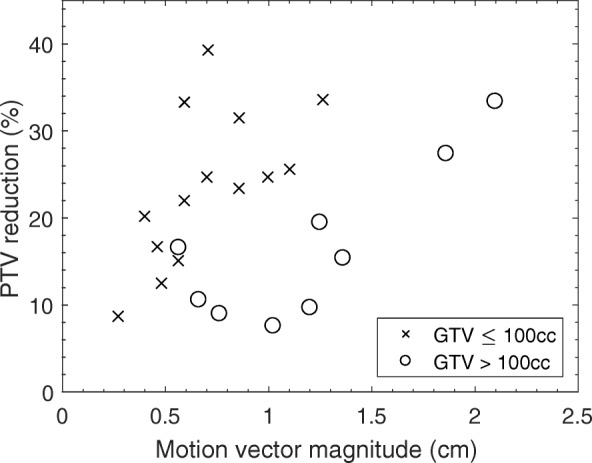
PTV reduction as a function of the magnitude of the motion vector of the centre of mass of the GTV. PTV reduction is presented as the difference in motion managed and ITV-based PTV sizes, as a percentage of the ITV-based PTV size

The reduction in PTV size as a result of eliminating the margin for motion is presented in Fig. 3. A monotonically increasing (Spearman’s rho 0.771, *p* = 0.002) correlation between the PTV size reduction and motion vector magnitude of the tumor centre of mass was observed for GTV sizes < 100 cm^3^. Cases with GTVs larger than this have been considered separately; as the GTV size increases, the volume becomes less spherical and more irregular, hence the direction of motion in relation to the largest dimension of the tumor dictates the volume of the associated PTV. This in turn reduces the correlation of motion vector magnitude to PTV reduction for the subset of large GTV sizes (Spearman’s rho 0.600, *p* = 0.097). The volume of liver no longer contained within the PTV by eliminating the margin due to motion ranged from 0.2–5% (2.4 cm^3^–77.1 cm^3^).

**Fig. 4:**
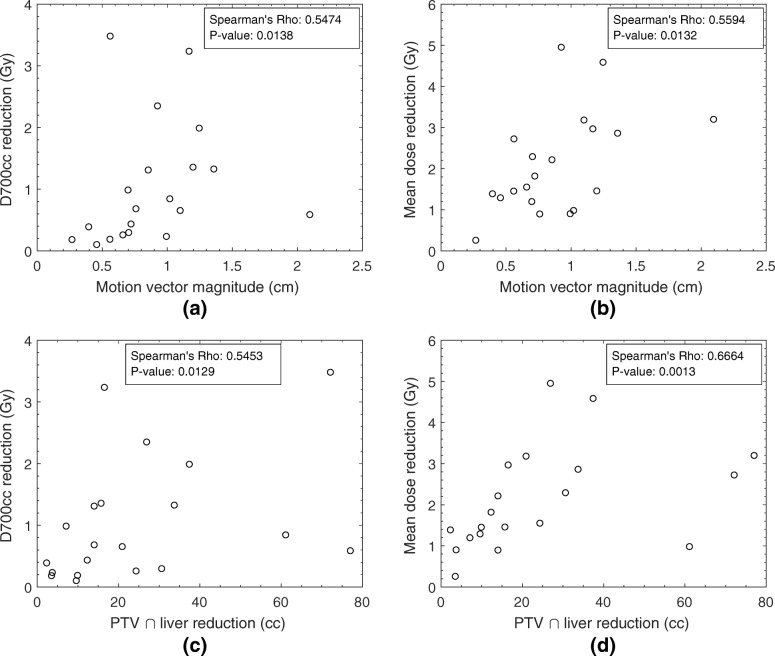
Reduction in the maximum liver dose to 700 cm^3^ and mean liver dose for the motion managed plan compared to the ITV-based plan, as a function of the magnitude of the motion vector of the GTV (parts (**a**) and (**b**)) and the liver volume spared from the PTV due to motion management (parts (**c**) and (**d**))

Figure 4 shows a decrease in the dose to the liver for the motion managed plan, compared to the ITV-based plan, as a consequence of the smaller target volume. A monotonically increasing relationship was observed between the two parameters used to evaluate liver toxicity, maximum dose to 700 cm^3^ and mean dose, and the magnitude of the motion vector (Spearman’s Rho 0.5474 and 0.5594 respectively), shown in parts (a) and (b). A similar correlation (Spearman’s Rho 0.5453 and 0.6664 respectively) is seen as the liver volume overlapping the PTV reduces for the motion managed PTV relative to the ITV-based PTV. The average reduction in mean dose to the surrounding liver was (2.1 ± 1.2) Gy across the patient cohort. The reduction in the maximal dose received by 700 cm^3^ of the surrounding liver was on average (1.0 ± 1.0) Gy.

### Dose escalation with motion management

**Fig. 5:**
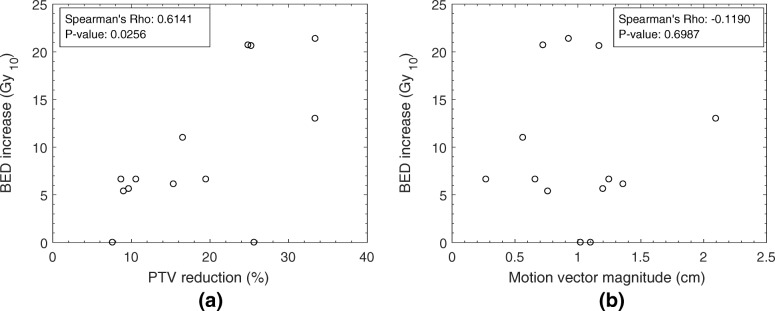
The relationship between dose escalation using the motion managed PTV, as a function of PTV reduction as a percentage of the ITV-based PTV (part (**a**)) and the motion vector magnitude of the GTV (part (**b**))

Figure 5 shows that for 11 of 13 patients where the initial prescribed dose was lower than the maximum dose level, tumor dose escalation was possible for the motion managed plan. The increase in BED ranged from 5.4 Gy_10_–21.4 Gy_10_. There was a strong correlation (Spearman’s Rho 0.6141, *p*-value 0.0256) between increasing escalation of dose and the magnitude of PTV reduction, shown in Fig. 5 part a. Poor correlation is seen between motion vector and BED increase, indicating that magnitude of motion alone should not be used as a predictor (see Fig. 5 part b). As shown in Fig. 3, for large GTVs (here defined as >100cm^3^) a large range of motion does not necessarily correspond to a large reduction in PTV volume.

The volume of the GTV itself can also be prohibitive to dose escalation. One of the two cases shown in Fig. 5 for which no BED increase was possible was due to a large GTV size of 1009 cm^3^. Despite a motion vector magnitude of 1.0 cm and a PTV volume reduction of approximately 100 cm^3^, the reduction in mean liver dose was not sufficient to allow dose escalation in this case.

**Fig. 6:**
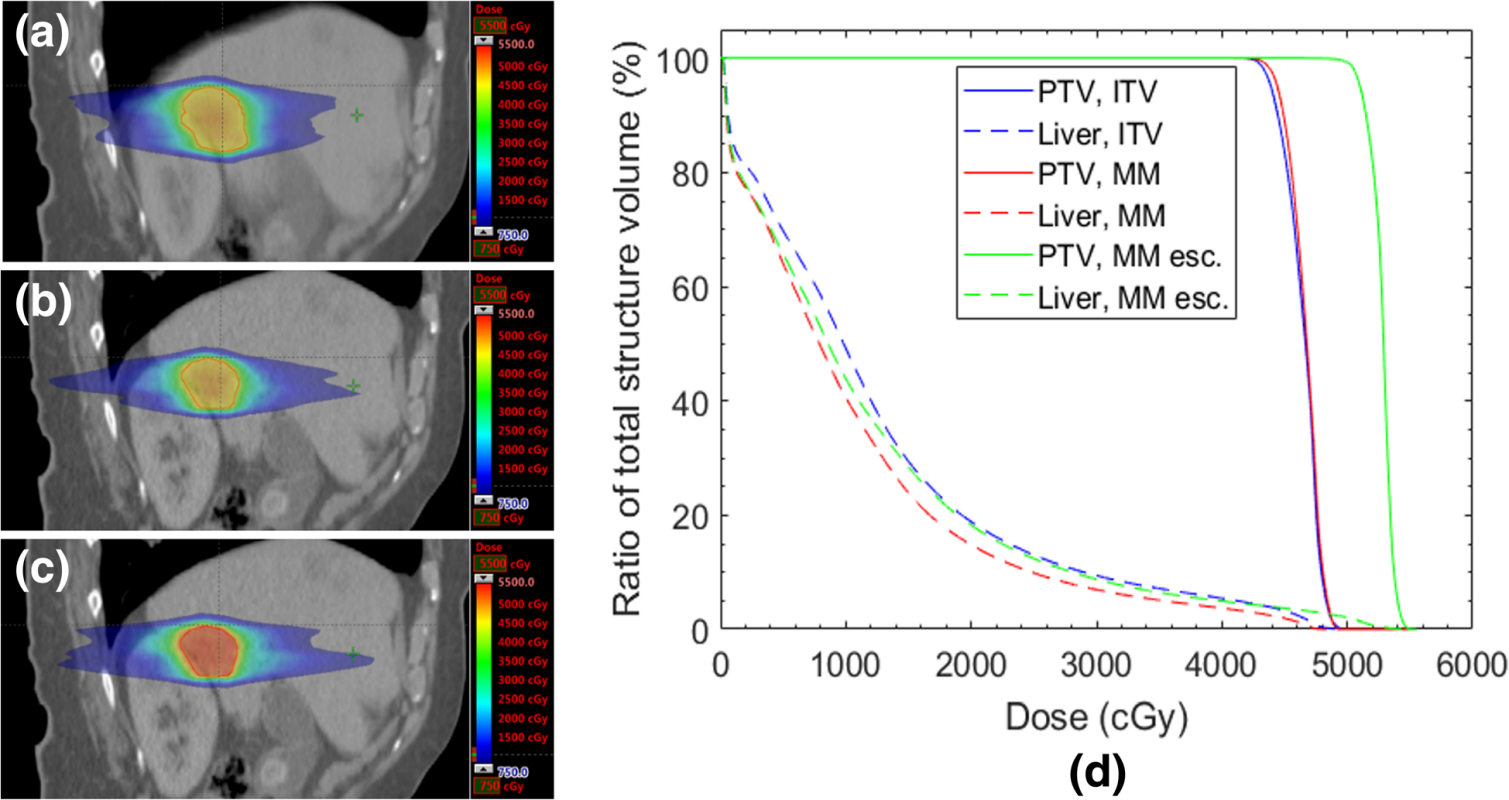
Part (**a**) shows the dose distribution to a lesion planned using the ITV method. Part (**b**) shows the re-plan using the motion managed PTV, at the same prescription level as in (**a**). Part (**c**) shows the escalation of dose, from 42.5 Gy to 50 Gy (78.6 Gy_10_ to 100 Gy_10_), whilst adhering to OAR dose tolerances. Part (**d**) is a DVH demonstating PTV coverage (solid lines) for the three cases shown in (**a**) – (**c**), as well as liver dose (broken lines). ITV – ITV-based, MM – motion managed, MM esc – dose escalated motion management

Figure 6 shows a comparison of dose distributions for a patient whose reduction in PTV size with motion management enabled the maximum dose level of 100 Gy_10_ to be prescribed without compromising OAR tolerances. For the ITV-based plan the maximum prescribed BED achieved without compromising OAR dose tolerance was 79.3 Gy_10_; this corresponds to a dose escalation of 20.7 Gy_10_. The ITV-based plan, the motion-managed plan and the dose-escalated motion-managed plan are shown in Fig. 6 parts (a) – (c) respectively. The mean dose to the liver remained similar to that of the ITV-based plan despite dose escalation to the PTV. With no dose escalation, the mean liver dose for the motion managed plan was 1.8 Gy lower than for the ITV-based plan.

Dose to the liver volume is not the sole prescription-limiting factor for SABR plans; proximity of other OAR structures can also prohibit dose escalation. For one of the cases shown in Fig. 5 no dose escalation was possible due to the proximity of the PTV to the chest wall, which was not reduced through motion management. Point maximum and volumetric dose constraints for the chest wall were not acceptable when the prescription dose was increased for the motion managed plan. Adjacent gastrointestinal structures such as the duodenum may also restrict the prescribed dose, as was observed for another case in this study. Despite a PTV reduction of 9% and motion vector of 0.8 cm, dose escalation with motion management was limited due to the persistent overlap of the PTV with the duodenum. The prescribed dose was unable to be increased beyond the maximum tolerable dose to 0.5 cm^3^ of the gastrointestinal structure, 30 Gy. These results provide context as to why a weaker correlation was seen between motion vector magnitude and BED increase (Fig. 5b), compared to motion vector magnitude and liver dose (Fig. 4). While a reduction in PTV size directly results in a reduction of treated liver volume, and hence liver dose, this does not translate to a potential for dose escalation in all cases. Dose escalation will only be possible if the introduction of motion management decreases the proximity of dose-limiting OARs.

### TCP gain from dose escalation

**Fig. 7:**
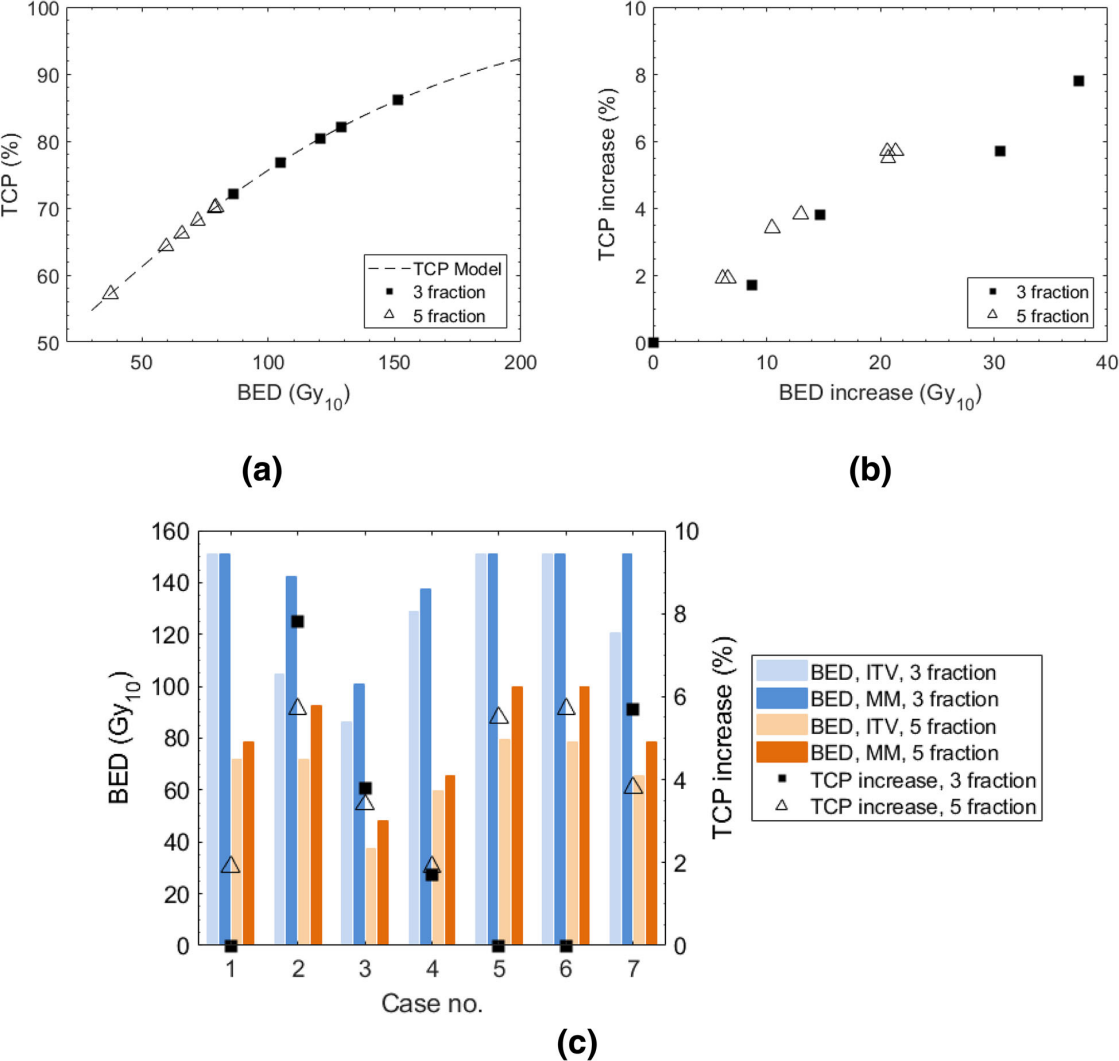
Comparison of TCP increase due to motion management (MM) for 3 and 5 fraction schedules. TCP is defined as the absolute increase in 24-month local control rate. Part (**a**) shows the prescribed BEDs (for the ITV-based plan) for the 3 and 5 fraction schedules, plotted alongside the HYTEC TCP model [14]. Part (**b**) plots the increase in TCP vs the corresponding BED increase for each fractionation schedule; the 3 fraction curve sits lower than the 5 fraction curve due to the decrease in the TCP curve gradient as BED increases. Part (**c**) shows the results per-patient, demonstrating that for the same anatomical geometry the BED increase ultimately determines which fractionation schedule has the highest increase in TCP, despite the change in the slope of the TCP curve

Figure 7a shows the position of each case on the TCP curve for the two fractionation schedules; the TCP corresponds to the prescribed BED for the ITV-based plan. In Fig. 7b, TCP gains for 3 fraction and 5 fraction prescription schedules are plotted against BED increases owing to motion management for the metastatic cohort of the study. The TCP gain due to motion management ranged from 1.9 to 5.7% for the 5 fraction prescription schedule. The TCP gain for the 3 fraction prescription schedule ranged from 0 to 7.8%. Figure 7b shows that the position on the TCP curve (shown in Fig. 7a) influences the BED increase required for the same percentage TCP increase, given the gradient of the TCP curve for the 5 fraction schedule is steeper than for the 3 fraction schedule (at the maximal BED). For example, it is shown that for a TCP increase of approximately 5%, a BED increase of 30 Gy_10_ is required for the 3 fraction schedule as compared with 20 Gy_10_ for the 5 fraction schedule. However, when directly comparing the fractionation schedules for the same geometry (i.e. by creating a plan for each fractionation schedule on the same patient), differences in the slope of the TCP curve did not dictate the result, as is shown in Fig. 7c. The absolute increase in BED was the overarching factor rather than the gradient, resulting in larger TCP increases for the 3 fraction schedule for cases 2, 3 and 7.

For cases 1, 5 and 6 no TCP increase is shown for the 3 fraction schedule because the maximum prescription level was able to be achieved for the ITV-based plan. In general, motion management was required in fewer cases for the 3 fraction schedule than the 5 fraction schedule to achieve the maximum prescribed dose, 151.2 Gy_10_ and 100 Gy_10_ respectively. For the three fraction schedule only one ITV-based plan did not achieve a BED of 100Gy_10_, at which there is an established relationship to improved local control.

## Discussion

The implementation of motion compensation techniques in liver SABR is expected to benefit a large number of patients, with approximately 40% of prescriptions being lowered in favour of avoiding normal tissue toxicity. The data presented shows quantitatively the perceived benefit, which is evident in cases for which mean liver dose is the prescription limiting factor. The results are intended to guide the formulation of patient selection criteria for the implementation of a motion management technique that facilitates a reduction, or ideally elimination, of the motion component of the PTV margin. Prior to simulation of the patient, the histology, GTV size and location are often the only known parameters. For the purposes of dose escalation, patients with a tumor volume less than 100 cm^3^ but greater than 20 cm^3^ are considered better candidates than those with larger GTVs. In this study, all GTVs with a volume less than 21 cm^3^ were planned to the top prescription level using the ITV-based method (motion range 0.5–1 cm), indicating that liver dose constraints in these cases are unlikely to be prescription-limiting. Up to a volume of 100 cm^3^, the PTV reduction increases more sharply with an increasing motion vector than for GTVs > 100 cm^3^, indicating greater potential for dose escalation in this range. It is important to note that this is not a rigid prerequisite; as was stated in the previous section, the location of the tumor in relation to other dose-limiting OARs, such as gastrointestinal structures and even the chest wall, is also a factor.

For all cases of dose escalation within the metastatic cohort, at least a 2% (absolute) increase in the TCP was observed, with a maximum of 7.8% increase for the 3# schedule and 5.7% for the 5# schedule. The magnitude of increase in BED, rather than the TCP curve gradient, was the overarching factor determining the TCP gains associated with each fractionation schedule. However, the results may be biased against differences in liver dose constraints applied between the schedules. Specifically, the RTOG1112 [21] and CORE [22] protocols specify for a 5 fraction prescription, the mean liver dose shall be below 13Gy; for the 3 fraction schedule the recommended constraints are for D_50%_ < 15 Gy and D_700cm_^3^ < 15 Gy. It is arguable that the 3 fraction constraints are easier to achieve. This is demonstrated by the fact that for three cases (cases 1, 5, and 6 in Fig. 7c) the ITV plan was able to be planned to the top prescription level, and that only one in seven of the metastatic cases did not achieve a BED of 100 Gy_10_ (the dose level correlated to local control, as discussed earlier) using the ITV plan. The results are a preliminary indication that motion management may be best utilised for the 50Gy/5# prescription to boost tumour dose to the 100 Gy_10_ level (at which there is an established relationship to improved local control), considering the additional resourcing required for motion management.

The method used to evaluate the clinical gains of the implementation of a motion management technique for liver SABR considers a non-descript strategy where there is zero motion contributed to the PTV margin. Depending on the motion management technique employed, there may be a requirement to increase the PTV margin to account for residual motions or inaccuracies in the motion monitoring strategy. This may affect dose sparing, and hence the capacity for dose escalation, gained through motion management. To estimate the impact of residual motion, the patient shown in Fig. 6 was replanned on the end-exhale image set using a PTV margin that accounts for motion over the 40–60% phases – that is, the phases neighbouring end-exhale. The magnitude of motion over the 40–60% phases was 2.5 mm. For context, the motion vector magnitude of the total respiratory cycle was 9 mm. Accounting for this motion using a 3-phase ITV margin, and maintaining the same 5 mm ITV-to-PTV expansion margin, the PTV volume increased by 1.5 cm^3^. When the motion-managed plan was re-created using the PTV inclusive of residual motion, the same magnitude of dose escalation (21.4 Gy_10_) was possible, while achieving the required OAR constraints.

Although out of the scope of the current work, 4D dose calculations could be performed to further elucidate the differences in liver dose for patients treated with and without motion management. This has been performed by Yeo et al [23]. and Jung et al. [24] for hypofractionated liver treatments, who showed 3–5% underestimation of mean liver dose for a 3D dose calculation on an average-intensity-projection CT dataset compared to a 4D dose calculation. It must be noted however that the mean liver dose criteria has not necessarily been derived from 4D dose calculation, therefore may not be valid to this scenario.

## Conclusions

A reduction in PTV size by removal of tumor motion was shown to directly correlate to the ability to escalate dose to the PTV in cases where dose to the surrounding liver volume was the prescription-limiting factor. The observed increase in BED was up to 21.4 Gy_10_ for a five fraction prescription schedule, and 37.5 Gy_10_ for a three fraction prescription schedule, corresponding the TCP gains of 5.7 and 7.8% respectively. Introduction of a motion management technique reduced the mean dose to the liver volume by (2.1 ± 1.2) Gy on average. Implementing a motion management technique that facilitates a reduction in the treated PTV volume is expected to increase the achievable BED in patients with GTVs in the 20–100 cm^3^ range in majority of cases.

## Data Availability

The datasets used and/or analysed during the current study are available from the corresponding author on reasonable request, except when individual privacy may be compromised.

## Abbreviations

BED: Biologically Effective Dose
GTV: Gross Tumor Volume
HyTEC: Hypofractionatioed Treatment Effects in the Clinic
ITV: Internal Tumor Volume
OAR: Organ At Risk
PTV: Planning Target Volume
RILD: Radiation Induced Liver Disease
SABR: Stereotactic Ablative Body Radiotherapy
TCP: Tumor Control Probability
VMAT: Volumetric Modulated Arc Therapy
